# The Research Progression and Clinical Significance of Circular RNAs in Head and Neck Cancers

**DOI:** 10.1155/2020/2712310

**Published:** 2020-10-21

**Authors:** Danhui Wang, Zhi Li, Yongjun Wu

**Affiliations:** ^1^Department of Pathology, Affiliated Xiangtan Hospital, University of South China, Xiangtan, Hunan Province, China; ^2^Department of Clinical Pharmacology, Xiangya Hospital, Central South University, Changsha, Hunan Province, China; ^3^Institute of Clinical Pharmacology, Central South University and Hunan Key Laboratory of Pharmacogenetics, Changsha, Hunan Province, China

## Abstract

With rapid development of science technique and molecular research, a large number of circular RNAs (circRNAs) were discovered. CircRNAs that are a heterogeneous endogenous group of non-coding RNA not only are abundantly and diffusely expressed in mammals but also participate in many biological processes, such as in tumor ingenuity and progress. CircRNAs have rarely open reports in the head and neck cancers (HNC), which are an aggressive malignant tumor with unsatisfactory overall survival rates. The diagnostics and treatments continue to improve while the survival rate of HNC patients has no more obvious improvement. Recent studies that are aimed at exploring the molecular mechanisms of occurrence and progression of circRNAs in HNC provide a valuable insight into potential novel diagnostic and therapeutic approaches. In this review, we summarize the increasing number of published researches on the research progression of circRNAs in HNC, as well as their possible clinical implications on HNC.

## 1. Introduction

Extensive reports have provided evidence that many non-coding RNAs (ncRNAs), such as microRNAs (miRNAs) and long ncRNAs (lncRNAs), can be applied to be potential biomarkers in early detection, early diagnosis, and early treatment [1, 2]. Recently, beyond that, through novel bioinformatic approaches and the RNA-seq technique, the role of circRNAs in HNC has been studied by researchers worldwide [3, 4]. CircRNAs, first discovered by electron microscopy in 1976 [5], were a subclass of ncRNAs that were thought to be continuous loops without the typical 3′ or 5′ ends [6]. And circRNAs were then found soon after in the cytoplasm of eukaryotic cells [7]. Compared to miRNAs and lncRNAs, circRNAs have their special biogenesis, properties, and biological functions. Due to the stability and tissue specificity of circRNA, various circRNAs have been demonstrated to regulate and influence cancers such as hepatocellular cancer [8], breast cancer [9], and colorectal cancer [10]. Head and neck cancers are the sixth most popular cancer worldwide [11] that occur in the oral cavity, thyroid gland, larynx, pharynx, and other sites. Low survival rates and mortality associated with head and neck cancers that remain a major healthcare burden globally are partly due to early diagnosis failure [12, 13]. So, precision diagnostic and therapeutic approaches are still urgently needed. The present review will provide a rough overview of circRNAs and highlight the most up-to-date research on the different circRNAs associated with HNC.

## 2. Circular RNAs

**Figure 1:**
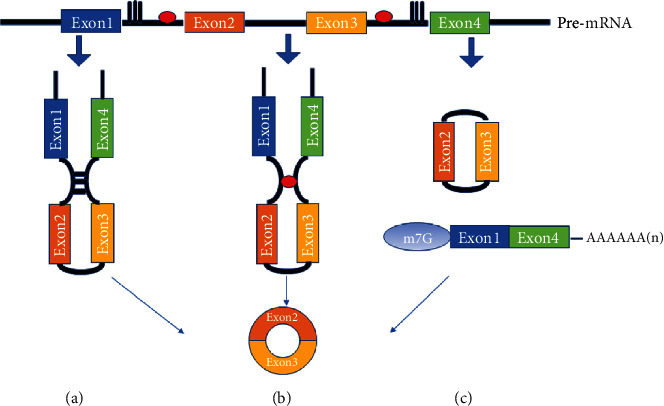
Models for the biogenesis of circRNAs. circRNAs are produced from special linear pre-mRNAs by back splicing circularization. (a) Introns which have complementary base pairs can contribute to the formation of circRNAs by bringing the adjacent two exons to close together. The exons and introns are then cut away by spliceosome to form circRNAs. (b) Both RNA binding proteins (RBP) and specific sequences in introns can serve as a bridge and stimulate back splicing circularization because they can dimerize and bring exons in close proximity. (c) That pair intron make exon skipping through back splicing produce a lariat precursor. Lariat precursors may also contribute to the formation of circRNAs.

CircRNAs are produced from special linear pre-mRNAs by back splicing circularization, and its formation can be divided into three categories (Figure 1). First, introns with orientation-opposite complementary sequences flanking circularized exons may be involved in the formation of circRNAs. These introns can contribute to the formation of circRNAs by pairing to form RNA duplexes that juxtapose the splice sites [14, 15]. Second, both RNA binding proteins (RBP) and specific sequences in introns can stimulate back splicing circularization because they can dimerize and bring exons in close proximity [16]. Third, related researches indicated that a lariat precursor, formed during exon skipping, may also contribute to the formation of circRNAs [17].

CircRNAs have several remarkable properties. First, circRNAs are not easy to be degraded by exonuclease for they do not have 5′ or 3′ tails [18]. Second, circRNAs are abundant. In some studies, circRNAs molecules are 10 times more than linear mRNAs [14]. Third, circRNAs are mainly composed of exons, primarily existing in the cytoplasm, and have miRNA response specificity [14, 19, 20]. Fourth, a large amount of circRNAs are endogenous ncRNAs, and only a small amount is exogenous circRNA [21]. All the above characteristics suggest that circRNAs play a vital role in transcription and translation and may be a biomarker for disease diagnosis.

Deeper mechanistic studies proved that circRNAs have different biological functions. (a) CircRNAs can function as miRNA sponges to regulate the effects of miRNA on gene expression. Wang and colleagues demonstrated that circ-DOCK1 contains potential binding sites for miR-196a-5p and decreases the activity of miR-196a-5p [22]. Through this mechanism, circ-DOCK1 increases BIRC3 expression. (b) CircRNAs can interact with RBPs to influence their functions. For example, Ashwal-Fluss and his team suggest that circ-Mbl harbor muscleblind (MBL) protein binding sites. When the two combined, it affects the biosynthesis of circ-Mbl [23]. (c) CircRNAs can function as regulators of parental gene expression. Some nucleus-localized circRNAs containing intronic sequences such as circular intronic ankrd52 (ciankrd52), circEIF3J, and circPAIP2 have been shown to regulate the transcription of their parental genes ANKRD52, EIF3J, and PAIP2 [24, 25]. (d) CircRNAs can encode some proteins or peptides. AbouHaidar has suggested that a circRNA (220 nt) was demonstrated to encode 16 kDa protein in the rice yellow mottle virus study [26]. Moreover, the fact that a peptide of 10 kDa can be translated by circ-Mbl1 was found in the synaptosome fractions of the fly brain [27].

## 3. Circular RNAs in Head and Neck Cancers

Recent researches have indicated that the quantity of circRNAs plays an increasingly important role in many cancers, including in HNC, showing that circRNAs could be ideal biomarkers [28]. CircRNAs are significantly enriched in tumor cell exosomes and are relatively stable in plasma and other biofluids [29] for they are not easy to be degraded by exonuclease. With the development of the research on HNC, many differentially expressed circRNAs promise to provide a novel sight about HNC. The sections below describe the dysregulated circRNAs implicated in HNC (Table 1).

### 3.1. Circular RNAs with Oral Cavity Cancer

Oral cavity cancer is one type of HNC which has a relatively poor prognosis. Oral cavity cancer consists of gingival cancer, tongue cancer, and lip cancer, and others. The main pathological type of oral cavity cancer is oral squamous cell cancer (OSCC). Many studies have indicated that some circRNAs, as miRNA sponges or regulators of parental gene expression, participate in the pathophysiological processes in the proliferation, progression, and apoptosis of OSCC. These research conclusions show that circRNAs play an important role in carcinogenesis of OSCC. Circ-0000140 is a circ-RNA derived from exons 7 to 10 of the KIAA0907 gene. Functionally, the expression of circ-0000140 can suppress cell proliferation, migration, and invasion, as well as facilitate cell apoptosis in vitro. Mechanistically, circ-0000140 enhances the expression of miR-31 and its target gene LATS2 by “sponging” miR-31, which suppresses the aggressive biological behavior of OSCC [30]. Su et al.'s study characterized hsa_circ_0005379 by using high-throughput transcriptome sequencing technology. More in-depth investigation revealed that hsa_circ_0005379 was downregulated in OSCC tissues, and cell lines, and functioned as an inhibitor of migration, invasion, OSCC growth, and proliferation. Mechanistically, hsa_circ_0005379 can regulate the expression level of the epidermal growth factor receptor (EGFR) pathway protein [31]. Zhu and colleagues investigated the relationship of hsa_circRNA_100533 and its target miRNA hsa-miR-933 [32]. They found that hsa_circRNA_100533 and hsa-miR-933 were down-regulated and up-regulated in OSCC tissues, respectively. And further works indicated that hsa_circRNA_100533 inhibited cell proliferation and migration and promoted cell apoptosis. Mechanistically, miR-933 is a downstream binding target of hsa_circRNA_100533, and miR-933 can target GNAS. Downregulated hsa_circRNA_100533 promotes miR-933 expression, which regulates GNAS expression. The study of Gao's group revealed that circ-PKD2, a sponge for miR-204-3p, was downregulated in OSCC tissues and cells. Functionally, overexpression of circ-PKD2 suppressed OSCC cell proliferation, migration, and invasion and induced apoptosis and cell cycle arrest [33]. Mechanistically, they pointed out that circ-PKD2 could regulate the expression of APC2 by the circ-PKD2/miR-204-3p/APC2 axis. Deng and colleagues recently found that the increased level of hsa_circRNA_043621 and the decreased level of hsa_circRNA_102459 were associated with cell proliferation, cell cycle G0/G1 phase arrest, and apoptosis. However, the specific mechanism of them requires further investigation. This finding revealed that circRNA_102459 and circRNA_043621 may play the role of tumor suppressor and promoter, respectively, in OSCC carcinogenesis [34].

Although surgery can cure most OSCC patients in the early stage, some terminal cancer patients lose the opportunity for surgery. The reason for this ending is the lack of precise and effective early diagnosis methods. The pathological biopsy is currently the gold standard for the diagnosis of oral cavity cancer, but this invasive examination is relatively painful for the patient, and the results require a long time and even longer if immunohistochemistry is required. CircRNAs play major roles in diagnosis and prognosis biomarker of OSCC. Deng et al.'s study revealed that hsa_circRNA_102459 and hsa_circRNA_043621 were significantly dysregulated in OSCC tissues by circRNA microarrays [34]. Functionally, clinical data analysis indicated that the expression of hsa_circRNA_102459 and hsa_circRNA_043621 were associated with the clinical stage, tumor differentiation, and lymph node metastasis. Hsa_circRNA_102459 and hsa_circRNA_043621 could be used as a new novel diagnosis and prognosis biomarker of OSCC. Besides, the expression level of hsa_circ_009755 was significantly low in OSCC tissues and cell lines compared with adjacent normal control and was related to the T stage of OSCC [35]. The area under the curve (AUC) of hsa_circ_009755 is 0.782, and the sensitivity and specificity are 70.37% and 77.78%, respectively. Therefore, hsa_circ_009755 could serve as a good prognosis biomarker of OSCC. CircBase analysis and qRT-PCR showed that hsa_circ_0072387 was downregulated in OSCC tissues compared with paired adjacent normal tissues and the human normal oral keratinocyte (hNOK) cell line [36]. Functionally, the hsa_circ_0072387 expression level was significantly related to the TNM stage of OSCC, and the AUC values of hsa_circ_0072387 were 0.746. These results suggested that hsa_circ_0072387 could serve as potential diagnostic biomarkers for OSCC.

CircRNAs can serve as regulators to participate in some cytological events in the development of OSCC, such as cell proliferation, migration, invasion, and apoptosis, which indicated that circRNAs have possibilities to be potential therapeutic targets for OSCC. Su et al.'s study proved that high hsa_circ_0005379 expression is related to tumor size and differentiation in OSCC patients and can obviously improve the sensitivity of OSCC to the cetuximab drug [31]. Functional experiments have confirmed that increased hsa_circ_0005379 obviously promotes the early apoptotic rate of OSCC cell lines. This indicates that the upregulation of hsa_circ_0005379 increases cetuximab sensitivity and provides a new potential target for OSCC anticancer drug design in the future. What is more, the study of Zhao's group revealed that circUHRF1 (hsa_circ_0002185) is markedly upregulated in OSCC samples compared with matched noncancerous tissues. Functionally, circUHRF1 positively correlates with epithelial-mesenchymal transformation (EMT), migration, invasion, and proliferation in vitro and the tumor growth in vivo [37]. Mechanically, circUHRF1 can firmly sponge miR-526b-5p to improve c-Myc expression, thereby promoting the progression of OSCC. This research sheds light on the possibility of circUHRF1 in the treatment of OSCC. Additionally, the fact that both circ-PKD2 and hsa_circRNA_100533 play a tumor-suppressive function in OSCC cells indicates that they may function as promising and effective therapeutic targets for OSCC [32, 33].

### 3.2. Circular RNAs with Papillary Thyroid Cancer (PTC)

Papillary thyroid cancer (PTC) is the most common histotype of thyroid cancer, which is the most prevalent endocrine malignancy worldwide [38]. However, despite its generally good prognosis and steadily ascended incidence, PTC can still threaten the quality of life of many patients due to invasiveness and metastasis [39]. The underlying and potential roles of circRNAs in PTC have been investigated in detail. Hsa_circ_0058124 locates on 2q35 in eukaryotic cells. Yao and colleagues found that hsa_circ_0058124 is significantly overexpressed in PTC cells. Functionally, they indicated that hsa_circ_0058124 promoted PTC cell proliferation, tumorigenicity, and tumor invasiveness both in vitro and in vivo [40]. Mechanically, hsa_circ_0058124 can abundantly sponge miRNA-218-5p to suppress the expression of NUMB, thereby regulating PTC cells through the NOTCH3/GATAD2A axis. Therefore, hsa_circ_0058124 could not only act as a potential biomarker for prognosis but also serve as a breakthrough point for new treatment and cancer screening in PTC patients.

Recent researches suggest that circFNDC3B (circ_0006156) expression is significantly dysregulated in several tumor types, such as bladder carcinoma [41] and gastric carcinoma [42], and circFNDC3B functions differ among these diseases. Wu and colleagues found that circFNDC3B expression suppresses the expression of miR-1178 and Toll-like receptor 4 (TLR4), suggesting that it can be a target for diagnosis and therapy [43]. Moreover, circFOXM1 (hsa_circ_0025033) is derived from FOXM1, and its length is 3410 nt. Ye et al. found high expression levels of circFOXM1 in PTC samples and correlates with the tumor growth of PTC in vitro and in vivo. Mechanistically, circFOXM1 functions as a competitive endogenous RNA (ceRNA) for miR-1179, thus activating the high-mobility group box 1 (HMGB1) signaling pathway to regulate the tumor growth of PTC. The above evidence suggests that circFOXM1 may provide hope for the treatment of PTC [44]. In a novel research of Cai's group, they found that circBACH2 was a tumor promoter in PTC, which modulated miR-139-5p as a sponge, thereby regulating the downstream gene LMO4 to suppress proliferation, migration, and invasion [45]. Liu and colleagues observed that levels of circRAPGEF5 are higher in PTC than in adjacent normal tissues. Functionally, circRAPGEF5 was beneficial to proliferation, migration, and invasion in vitro [46]. Mechanismally, circRAPGEF5 affected PTC progression by serving as a miR-198 sponge and regulating fibroblast growth factor receptor 1 (FGFR1) expression. These studies indicated that circRAPGEF5 acts as an oncogene via a novel circRAPGEF5/miR-198/FGFR1 axis, providing a potential biomarker and therapeutic target for the management of PTC.

### 3.3. Circular RNAs with Laryngeal Squamous Cell Cancer (LSCC)

In the world, ~30% of malignancies among HNC are laryngeal cancer (LC) [47]. Laryngeal squamous cell cancer (LSCC) is the most common type of LC with a relatively high mortality rate and poor survival rate [48]. The current standard treatment strategies have significantly improved the quality of life of LSCC patients, and the overall five-year survival rate remains unsatisfactory [49]. Some researchers discovered that circRNAs may provide a novel sight to LSCC. Tian et al.'s study analyzed differentially expressed circRNAs in three paired LSCC tissues and adjacent normal tissues by microarray analysis, among which 527 were upregulated and 414 were downregulated in LSCC tissues compared with normal tissues [50]. The upregulated circRNA with more than 4-fold change was hsa_circ_0059354, which is located on chromosome 20 and derived from RASSF2. Functionally, circRASSF2 knockdown decreased cell proliferation and migration. Their current work revealed that the circRASSF2 was an oncogenic factor that promoted tumorigenesis by serving as a ceRNA to regulate IGF1R expression by sponging miR-302b-3p. CircRASSF2 maybe a potential biomarker for the diagnosis and prognosis of LSCC, and further underlying mechanisms are needed to be investigated in the future. Based on the works of Tian et al.'s group [50], Wei and colleagues found that the expression level and function of hsa_circ_0042666 in LSCC were lower compared to adjacent nontumor tissues [49]. Functionally, they proved that the down-regulation of hsa_circ_0042666 is related to advanced tumor stage, lymph node metastasis, and poor overall survival in LSCC patients. A mechanism study demonstrated that hsa_circ_0042666 could regulate TGFBR3 expression by sponging miR-2238, then significantly influencing LSCC cell proliferation and invasion in vitro. This study might provide a precise therapeutic strategy for LSCC patients. In addition, Zhang et al.'s study revealed that ciRS-7 (CDR1as) is differently expressed in 30 paired LSCC tissues and adjacent normal tissues [51]. High CDR1as levels were demonstrated to relate to high TNM stages, poorly differentiated tumors, lymph node metastases, and poor prognosis of LSCC patients. It can enhance cell vitality and promoted the proliferation and migration via regulation of the miR-7/CCNE1/PIK3CD signaling pathway. CDR1as is an oncogene that promotes LSCC progression.

### 3.4. Circular RNAs with Others

CircRNAs have also been studied in other head and neck cancers. Epstein-Barr virus (EBV) is an etiological factor for nasopharyngeal cancer (NPC) which is prevalent in Asia [52]. In a recent study, Chen et al.'s group screened that circRNA_000543 was higher in NPC samples than in adjacent tissues [53] and was associated with poorer overall survival. Deeper researches revealed that circRNA_000543 increased NPC cell sensitivity by targeting the miR-9/platelet-derived growth factor receptor B (PDGFRB) axis. CircRNA_000543 may be a potential therapeutic target for NPC patients who are radioresistant. For hypopharyngeal squamous cell cancer (HSCC), research on circRNAs was first conducted in 2017 by Cao and colleagues [54]. These researchers analyzed that hsa_circ_0058106 and hsa_circ_0036722 were differentially expressed in 4 pairs of HSCC tissues and normal controls by using a circRNA microarray. Hsa_circ_0058106 and hsa_circ_0036722 were significantly increased and decreased in tumor tissues compared with adjacent normal tissue samples, respectively. These data agree with the results of the microarray data. Bioinformatic analysis of the deregulated hsa_circ_0036722, bond to miR-671-5p, might facilitate the invasiveness of HSCC. Hsa_circ_0058106 might sponge miR-185-3p and then prevent HSCC cells from undergoing apoptosis. However, there are many works to uncover the underlying mechanisms of circRNAs in HSCC. Mucoepidermoid carcinoma (MEC) of the salivary gland is a malignant neoplasm which has high probability of distant metastasis and poor prognosis. However, the underlying pathogenesis of MEC is still little known. Yang and colleagues use microarrays to simultaneously detect the expression level of mRNAs, lncRNAs, and circRNAs in four pairs of MEC and matched normal tissues. A total of 3612 mRNAs, 3091 lncRNAs, and 284 circRNAs were dysregulated [55]. Gene Ontology (GO) and Kyoto Encyclopedia of Genes and Genomes (KEGG) analysis were used to predict the functions of these differentially expressed RNAs. qRT-PCR analysis was used to further screen and confirm NONHSAT154433.1, associated with ADAM12, and hsa_circ_0012342. In conclusion, NONHSAT154433.1 and hsa_circ_0012342 could act as potential prognostic biomarkers and therapeutic targets of MEC.

## 4. Conclusion and Prospects

HNC is a multistep and multifactor comprehensive disease, and the specific biogenesis, development, and treatment are still not fully understood. CircRNAs can significantly affect many different biological processes of HNC, such as cell proliferation, migration, invasion, drug resistance, and apoptosis. CircRNAs are considered to be miRNA sponges and parental gene transcriptional regulators which may participate in cancer occurrence and progression through a complex set of mechanisms. CircRNAs could be applied to clinical practice for their dysregulated expression which is associated with the clinical stage, tumor stage, lymph node metastasis, and the prognosis of HNC patients.

CircRNAs are significantly enriched in tumor tissues and are relatively stable in plasma and other biofluids because of their several remarkable properties that are mentioned above. Similar to lncRNAs and microRNAs, quantitative real-time polymerase chain reaction (qRT-PCR) is mainly applied to quantification circRNAs. Microarray technology and deep sequencing are adopted for high-throughput analysis of clinical samples and verify the results, respectively. In a word, circRNAs, as novel and reliable biomarkers, play important roles in the diagnosis, prognosis, and treatment of HNC. However, compared with other noncoding RNAs, such as lncRNAs and miRNAs, our current researches of circRNAs are still inadequate. There are some suggestions for future circRNAs research. First, we should further investigate the novel HNC-relevant circRNAs that researchers identified in their study to explore the specific HNC signaling pathway(s) they are involved in, because the data provided by databases and prediction tools need to be verified. Second, recent researches on circRNAs in cancers mainly use tumor cells and tumor tissue samples. More cell lines, animal studies, clinical studies, and noninvasive samples (blood, urine, saliva, etc.) would be further required to be used for research and detection. Third, we should increase the collection of clinical information about samples, such as sex, age, location, and pathological staging. This will help us to further evaluate the relationship between HNC-relevant circRNAs and diagnosis, prognosis, and treatment of patients with OSCC. Fourth, future works should solve the problem of how to transport circRNAs to relevant sites for blocking the proliferation of cancer cells and how to reduce cancer cell resistance. Fifth, unconventional functions of circRNAs, such as translation and protein binding, should be further explored. Sixth, there are problems that need to be overcome, for example, detecting circRNAs in human tissues or in biofluids is more money-consuming and time-consuming than existing tumor protein markers, such as carcinoembryonic antigen (CEA) and cancer antigen 19–9 (CA19–9). With the rapidly improving RNA sequencing technologies, as well as the appearance of more advanced bioinformatic tools, we believe that the detection and analysis of relevant circRNAs will become more practical and cost-effective. And circRNAs will be studied more and bring great progress to the diagnosis, treatment, and prognosis of HNC patients.

## Figures and Tables

**Table 1:** Dysregulated circRNAs in HNC.

Primary site	Name	Gene symbol	Dysregulation	Sponge target	Function	Clinical value	Possible mechanism	Reference
OSCC	hsa_circ_0000140	LATS2	Down	miR-31	Suppress cell proliferation, migration, and invasion and facilitated cell apoptosis in vitro	A promising prognostic biomarker and potential treatment strategy for OSCC	circ_0000140/miR-31/LATS2 axis	[30]
	hsa_circ_0005379		Down		Inhibits cancer cell proliferation, invasion	A new therapeutic target for OSCC treatment	Regulate the epidermal growth factor receptor (EGFR) pathway	[31]
	hsa_circRNA_100533	GNAS	Down	miR-933	Inhibit cell proliferation and migration, promote cell apoptosis, and regulate GNAS expression	An effective diagnostic biomarker and therapeutic target for patients with OSCC	hsa_circRNA_100533-miR-933-GNAS axis	[32]
	circ-PKD2	APC2	Down	miR-204-3p	Inhibit OSCC cell proliferation, migration, and invasion, induce apoptosis and cell cycle arrest	A novel pathway involved in the pathogenesis of OSCC and may serve as a novel therapeutic target of OSCC	circ-PKD2/miR-204-3p/APC2 axis	[33]
	hsa_circRNA_102459		Down		Relate to clinical stage, tumor differentiation, and lymph node metastasis and suppressed TSCC1 cell proliferation, induced cell cycle G0/G1 phase arrest, and promoted apoptosis	A tumor promoter and valuable diagnostic biomarkers of OSCC		[34]
	hsa_circRNA_043621		Up		Relate to clinical stage, tumor differentiation, and lymph node metastasis and suppressed TSCC1 cell proliferation, induced cell cycle G0/G1 phase arrest, and promoted apoptosis	A tumor promoter and valuable diagnostic biomarkers of OSCC		[34]
	hsa_circ_009755		Down		Associated with T stage of OSCC	A potential biomarker for OSCC diagnosis		[35]
	hsa_circ_0072387		Down		Related to TNM stage of OSCC	A potential diagnostic biomarker		[36]
	circUHRF1	UHRF1	Up	miR-526b-5p	Associated with proliferation, migration, invasion, and epithelial-mesenchymal transformation (EMT) in vitro and the tumor growth in vivo	A potential therapeutic target	circUHRF1/miR-526b-5p/c-Myc/TGF-*β*1/ESRP1 feedback loop	[37]
PTC	hsa_circ_0058124	NUMB	Up	miR-218-5p	Promotes PTC cell proliferation, tumorigenicity, and tumor invasiveness both in vitro and in vivo	A novel biomarker and a novel therapeutic target for intervening in PTC progression	hsa_circ_0058124/NOTCH3/GATAD2A axis	[40]
	hsa_circ_0006156	FNDC3B	Up	miR-1178	Knockdown of circFNDC3B inhibited cell proliferation and promoted cell apoptosis in PTC cells	A promising therapeutic target for the treatment of PTC patients	hsa_circ_0006156/miR-1178/TLR4 pathway	[43]
	circFOXM1	FOXM1	Up	miR-1179	circFOXM1 downregulation inhibited tumor growth of PTC in vitro and in vivo	A promising therapeutic target for the treatment of PTC patients	circFOXM1/miR-1179/high-mobility group box 1 (HMGB1) axis	[44]
	circBACH2	LMO4	Up	miR-139-5p	Suppress proliferation, migration, and invasion	A promising prognostic biomarker and potential treatment strategy for PTC	circBACH2/miR-139-5p/LMO4 axis	[45]
	circRAPGEF5	RAPGEF5	Up	miR-198	Beneficial to proliferation, migration, and invasion in vitro	A potential biomarker and therapeutic target for the management of PTC	circRAPGEF5/miR-198/FGFR1 axis	[46]
LSCC	hsa_circ_0059354	RASSF2	Up	miR-302b-3p	Silencing circRASSF2 suppresses progression of LSCC by interacting with miR-302b-3p and decreasing inhibiting IGF-1R expression	A potential biomarker for the diagnosis and prognosis of LSCC	hsa_circ_0059354/miR-302b-3p/IGF-1R axis	[50]
	hsa_circ_0042666	TGFBR3	Down	miR-2238	Associated with advanced tumor stage, lymph node metastasis, and poor overall survival in LSCC patients	A promising target for LSCC treatment and a tumor suppressor	hsa_circ_0042666/miR-223/TGFBR3 axis	[49]
	ciRS-7	CDR1as	Up	miR-7	Relate with high TNM stages, poorly differentiated tumors, lymph node metastases and poor prognosis of LSCC patients. Enhance cell vitality and promoted the proliferation and migration	An oncogene that promotes LSCC progression	miR-7/CCNE1/PIK3CD signaling pathway	[51]
NPC	circRNA_000543	PDGFRB	Up	miR-9	Associated with poorer overall survival in NPC patients	A potential therapeutic target for radioresistant NPC	miR-9/platelet-derived growth factor receptor B (PDGFRB) axis	[53]
HSCC	hsa_circ_0058106		Up	miR-185-3p	Prevent HSCC cells from undergoing apoptosis	Provide potential candidates for future mechanism studies		[54]
	hsa_circ_0036722	RHCG	Down	miR-671-5p	Facilitate the invasiveness of HSCC	Provide potential candidates for future mechanism studies		[54]
MEC	hsa_circ_0012342		Down		Closely related to the pathogenesis of MEC	Potential prognostic biomarkers and therapeutic target of MEC		[55]

Dysregulated circRNAs in HNC. Abbreviations: OSCC: oral squamous cell cancer; PTC: papillary thyroid cancer; LSCC: laryngeal squamous cell cancer; NPC: nasopharyngeal cancer; HSCC: hypopharyngeal squamous cell cancer; MEC: mucoepidermoid carcinoma.
